# Draft genome sequence of *Pseudomonas aeruginosa* SAU_MI_2M5 isolated from milk of cattle in Dhaka, Bangladesh

**DOI:** 10.1128/mra.00691-26

**Published:** 2026-07-28

**Authors:** Arpita Biswas, Md Hafizur Rahman, Md. Mominul Islam, Alich Mondal, Rahnuma Tabassum Taqwa, Fatema Tuj Zohura, Md. Rashedul Islam, Mahfuzul Islam

**Affiliations:** 1 Department of Microbiology and Parasitology, Sher-e-Bangla Agricultural University 54494, Dhaka, Bangladesh; 2 AMR Reference Laboratory (Research), Animal Health Research Division, Bangladesh Livestock Research Institute 560480, Dhaka, Bangladesh; 3 Department of Pathology, Sher-e-Bangla Agricultural University 54494, Dhaka, Bangladesh; 4 Department of Surgery and Theriogenology, Sher-e-Bangla Agricultural University 54494, Dhaka, Bangladesh; Portland State University, Portland, Oregon, USA

**Keywords:** *Pseudomonas aeruginosa*, cattle, milk, antibiotic resistance genes

## Abstract

A draft genome sequence of *Pseudomonas aeruginosa* isolated from the milk of cattle in Dhaka, Bangladesh, is described in this report. The 7.24 Mb genome was sequenced using the Illumina NextSeq platform and found to encode 6,958 predicted coding sequences, including putative antibiotic resistance genes.

## ANNOUNCEMENT

*Pseudomonas aeruginosa* is a versatile opportunistic pathogen commonly found in environmental and animal reservoirs and noted for its high adaptability and multidrug-resistant nature. This bacterium can cause diverse infections and represents a major concern for both animal and human health (1, 2).

A draft genome sequence of *P. aeruginosa* obtained from an apparently healthy feedlot dairy cow in Dhaka, Bangladesh in 2025 is reported here. Raw milk was aseptically obtained from a dairy cow and transferred to 9 mL of peptone water for enrichment (3). After aerobic incubation at 37°C for 6 h, the enriched culture was streaked onto nutrient agar (HIMEDIA, India) and incubated for an additional 20 h at 37°C. An isolated pure colony was chosen for downstream characterization, and species identity was confirmed by matrix-assisted laser desorption/ionization time-of-flight (MALDI-TOF) mass spectrometry (4). The identified *P. aeruginosa* was then considered for whole-genome sequencing (WGS).

Genomic DNA was extracted from an overnight nutrient broth culture incubated at 37°C using the QIAamp DNA Mini Kit (QIAGEN, Germany). WGS was performed on the Illumina NextSeq 2000 platform (Illumina, San Diego, CA, USA) using a paired-end (2 × 150 bp) strategy, generating 6,695,396 pair-end reads. Sequencing libraries were prepared with the Nextera XT Library Preparation Kit (Illumina, USA). Raw sequencing data were evaluated for quality using FastQC v0.74 (5), and adapter sequences along with low-quality bases were removed using Trimmomatic v0.39 with default parameters (6). Following quality control and trimming, a total of 6,683,664 high-quality reads were retained for downstream genome assembly. Using Unicycler v0.4.8 with default parameters (7), *de novo* assembly generated a draft genome containing 57 contigs with a mean sequencing coverage of 117.22×. Genome completeness was assessed using BUSCO v5.8.0 (8) in prokaryotic genome mode with the pseudomonadales_odb10 lineage data set. The draft genome has a total length of 7,236,130 bp, a guanine-cytosine (GC) content of 64.97%, and an N50 value of 435,632 bp. The draft genome assembly showed 98.8% completeness (98.6% single-copy, 0.3% duplicated), with 0.1% fragmented and 1.0% missing BUSCO genes. Genome annotation was performed using the National Center for Biotechnology Information (NCBI) Prokaryotic Genome Annotation Pipeline (PGAP) v6.10 (9), which identified 6,958 protein-coding sequences, 3 rRNA genes, and 103 tRNA genes. Further characterization of the assembled genome using AMRFinderPlus v3.11.20 (10) and ResFinder v4.7.2 (11, 12) detected nine putative antimicrobial resistance (AMR) genes, including *oprD_V359L*, *aph(3′)-Iib*, *blaOXA-486*, *blaPDC-121*, *blaPAO*, *catB7*, *tmexD2*, *crpP*, and *fosA*. Analysis with CRISPRimmunity (13) identified a single CRISPR array within the bacterial genome. Analysis using PAst (13), PathogenFinder2 (14), and MLST v2.0 (15) predicted the isolate to belong to serotype O9, identified it as a potential pathogen to humans with a probability score of 0.753, and assigned it to the sequence type (ST) 164, respectively. Metabolic functional annotation was performed using RAST v2.0 (16), which identified 397 subsystems with 25% subsystem coverage and a total of 1,760 associated genes. Default parameters were applied for all analyses unless stated otherwise.

## Data Availability

The sequence reads are deposited in the Sequence Read Archive under accession number SRR36966175 and the assembled genome under accession number JBUCWJ000000000. The associated BioProject and BioSample accession numbers are PRJNA1412526 and SAMN54812904, respectively. The output files from AMRFinderPlus, ResFinder, PathogenFinder, Serotype, RAST, and MLST analyses have been made publicly accessible via Figshare at the following link: https://figshare.com/s/fc0a1ddfda8911d87fb7.
